# Botanical-Based Strategies for Controlling *Xanthomonas* spp. in Cotton and Citrus: In Vitro and In Vivo Evaluation

**DOI:** 10.3390/plants14060957

**Published:** 2025-03-19

**Authors:** Roxana Andrea Roeschlin, María Alejandra Favaro, Bruno Bertinat, Fernando Gabriel Lorenzini, Marcelo Javier Paytas, Laura Noemí Fernandez, María Rosa Marano, Marcos Gabriel Derita

**Affiliations:** 1 Instituto Nacional de Tecnología Agropecuaria (INTA), Estación Experimental Agropecuaria (EEA) Reconquista, Ruta 11 km 773 (S3560), Reconquista 3560, Argentina; roeschlin.roxana@inta.gob.ar (R.A.R.); lorenzini.fernando@inta.gob.ar (F.G.L.); paytas.marcelo@inta.gob.ar (M.J.P.); 2 Consejo Nacional de Investigaciones Científicas y Tecnológicas (CONICET), Godoy Cruz 2290, Argentina; 3 ICiAgro Litoral (UNL-CONICET), Kreder 2805, Argentina; mfavaro@fca.unl.edu.ar (M.A.F.); laurafernandez1@gmail.com (L.N.F.); 4 Facultad de Ciencias Agrarias, UNL, Kreder 2805, Argentina; brunobertinat97@gmail.com; 5 Instituto de Biología Molecular y Celular de Rosario (IBR), FCByF-UNR-CONICET, Área Virología, Ocampo y Esmeralda S/N, S2002LRK, Rosario 2000, Argentina; marano@ibr-conicet.gov.ar; 6 Farmacognosia, FCByF-UNR, Suipacha 531, Rosario 2000, Argentina

**Keywords:** cotton bacterial blight, citrus canker, natural products, antibacterial activity, *Schinus*, *Persicaria*, *Pelargonium*

## Abstract

Citrus canker, caused by *Xanthomonas citri* subsp. *citri*, and bacterial blight, caused by *Xanthomonas citri* subsp. *malvacearum*, results in substantial economic losses worldwide, and searching for new antibacterial agents is a critical challenge. In this study, regional isolates AE28 and RQ3 were obtained from characteristic lesions on *Citrus limon* and *Gossypium hirsutum*, respectively. Essential oils extracted by steam distillation from the fresh aerial parts of *Pelargonium graveolens* and *Schinus molle* exhibited complete (100%) inhibition of bacterial growth in vitro at a concentration of 1000 ppm, as determined by diffusion tests. To evaluate the potential of these essential oils for controlling *Xanthomonas*-induced diseases, in vivo assays were conducted on lemon leaves and cotton cotyledons inoculated with the regional AE28 and RQ3 strains. Two treatment approaches were tested: preventive application (24 h before inoculation) and curative application (24 h after inoculation). Preventive and curative treatments with *P. graveolens* essential oil significantly reduced citrus canker severity, whereas *S. molle* essential oil did not show a significant reduction compared to the control. In contrast, regardless of the treatment’s timing, both essential oils effectively reduced bacterial blight severity in cotton cotyledons by approximately 1.5-fold. Gas chromatography–mass spectrometry (GC-MS) analysis identified geraniol and citronellol as the major components of *P. graveolens* essential oil, while limonene and t-cadinol were predominant in *S. molle*. These findings highlight the promising potential of botanical products as bactericidal agents, warranting further research to optimize their application and efficacy.

## 1. Introduction

The production of citrus fruits and cotton plays a crucial role in the global economy, providing essential resources for food, textiles, and numerous industries while supporting millions of jobs worldwide. According to the United States Department of Agriculture, citrus is the world’s most economically significant fruit tree crop, with a global production of 103.7 million metric tons in the 2023/2024 season [1]. Cotton (*Gossypium hirsutum* L.), often called “white gold” due to its economic importance, is the world’s leading natural textile fiber, cultivated on approximately 32.2 million hectares globally, driving an industry worth over USD 600 billion annually [2]. In Argentina, citrus and cotton are vital agricultural commodities, forming the backbone of regional economies with substantial economic and social impact [3,4].

Bacterial diseases caused by *Xanthomonas* spp. represent a continuous threat to citrus and cotton crops, leading to substantial reductions in production in all growing areas around the world. The bacteria adopt different lifestyles and strategies for virulence and dispersion in both hosts, showing variability in their tissue-specific invasion and causing different symptoms [5]. *Xanthomonas citri* subsp. *citri* (*X. citri*), a non-vascular pathogen, causes citrus bacterial canker, which can drastically reduce crop yields by leading to defoliation in susceptible cultivars and triggering premature fruit drop [6]. On the other hand, *X. citri* subsp. *malvacearum* (*X. malvacearum*), a vascular pathogen, is the causative agent of cotton bacterial blight, a high-risk pathogen for crop production, by inducing several symptoms including angular leaf spots, stem black arm, and boll rot [7]. Cultural practices, genetic strategies, and chemical control are key components of integrated management systems in countries where these bacteria are endemic. The strategies primarily involve selecting cultivars with improved tolerance and the application of chemical control, such as copper sprays in citrus plants and acid-delinting of cotton seeds, to reduce bacterial spread [8,9,10,11,12].

Despite the significant progress made in managing citrus and cotton bacterial diseases, both pathogens have evolved and reemerged in crop fields as new variant strains or copper-resistant *Xanthomonas* populations, constantly threatening crop yields [10,13,14,15,16,17]. In this context, natural plant products offer structurally different molecules that are selectively active against various phytopathogen species, biodegradable, and suitable for use as control agents in integrated management programs to complement commercial bactericides [18,19]. Numerous studies have demonstrated that plant extracts and essential oils exhibit antimicrobial activity against *Xanthomonas* species [11,20,21,22]. Previous research conducted in our laboratory has shown that plant extracts and essential oils from *Persicaria acuminata* (*P. acuminata*), *Schinus molle* (*S. molle*), and *Pelargonium graveolens* (*P. graveolens*) possess antifungal activity against various phytopathogens [18,23,24,25,26]. Building on these findings, this study aimed to evaluate these natural products in vitro and in vivo activity against *X. citri* and *X. malvacearum*. So, the selection of these three plant species for this work was based on our hypothesis that, if they were useful to control fruit diseases caused by different phytopathogens, they could probably act as bactericides against *Xanthomonas* also [18,23,24,25,26]. Among these three, *P. graveolens* and *S. molle* produce volatile compounds that can be obtained by steam distillation in the form of essential oils, whereas *P. acuminata* does not biosynthesize volatile compounds and the preparation of fixed extract from its dry leaves is suitable [23,24,25]. Additionally, we selected a concentration of 1000 ppm for the tested natural products because, as observed in previous studies [23,24,25,26], lower concentrations failed to inhibit microorganisms in in vivo tests, while higher concentrations would not be practical for commercial applications.

## 2. Results

### 2.1. Plant Species Data Collection and Natural Product Yields

Table 1 presents data on plant material collection and natural product yields. For *P. acuminata*, it shows the yield of dry ethyl acetate (EtOAc) extract (grams of dry extract per 100 g of dried leaves). For essential oils, it indicates the volume (in milliliters) of product obtained from 100 g of freshly harvested aerial parts of each species.

### 2.2. Characterization of Xanthomonas citri Strains

**Figure 1:**
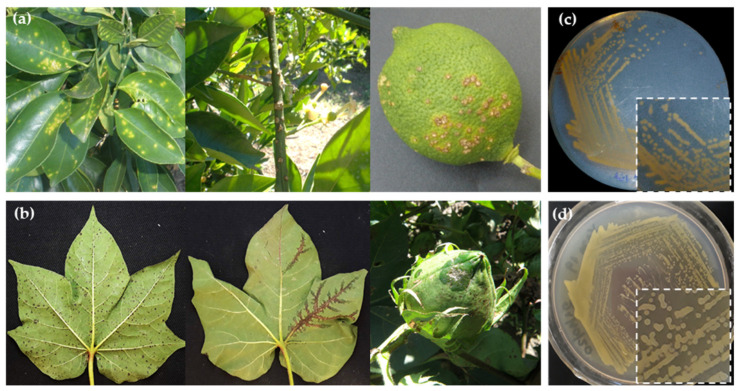
Symptomatology and morphological characterization of the regional strains *X. citri* subsp. *citri* and *X. citri* subsp. *malvacearum*. (**a**) Necrotic lesions surrounded by oily, water-soaked margins in leaves, branches, and fruits of *C. limon*. (**b**) Typical angular water-soaked lesions in cotton leaves, extending along the vascular system and in bolls. *X. citri* AE28 (**c**) and *X. malvacearum* RQ3 (**d**) colonies grown on a NYGB-agar plate. Bacterial colonies are shown enlarged at the bottom inset.

Regional *X. citri* (named AE28) and *X. malvacearum* (named RQ3) isolates were obtained from typical lesions *C. limon* (Figure 1a) and *G. hirsutum* (Figure 1b) Argentinean crops and used in this work. Both strains showed typical *Xanthomonas* spp. morphological characteristics, such as yellow, convex, and mucoid colonies (Figure 1c,d). The identity of AE28 strain was corroborated as *X. citri* subsp. *citri* using the *xpsD* genome-specific marker (Supplementary Materials, Figure S1). To confirm *X. malvacearum* RQ3 strain, PCR amplification of five *Xanthomonas*-specific housekeeping genes (*lepA*, *gapA*, *fusA*, *gltA*, *lacF*) was conducted. Sequenced fragments and BLASTn searches revealed that strain RQ3 shared 100% sequence similarity to previously characterized *X. malvacearum* gene sequences available in the NCBI database. The nucleotide sequences were deposited in GenBank (PV091809 to PV091813). Other reference strains used in this study are described in Table 2.

### 2.3. Effects of Natural Plant Products on Xanthomonas Strains Growth In Vitro

**Figure 2:**
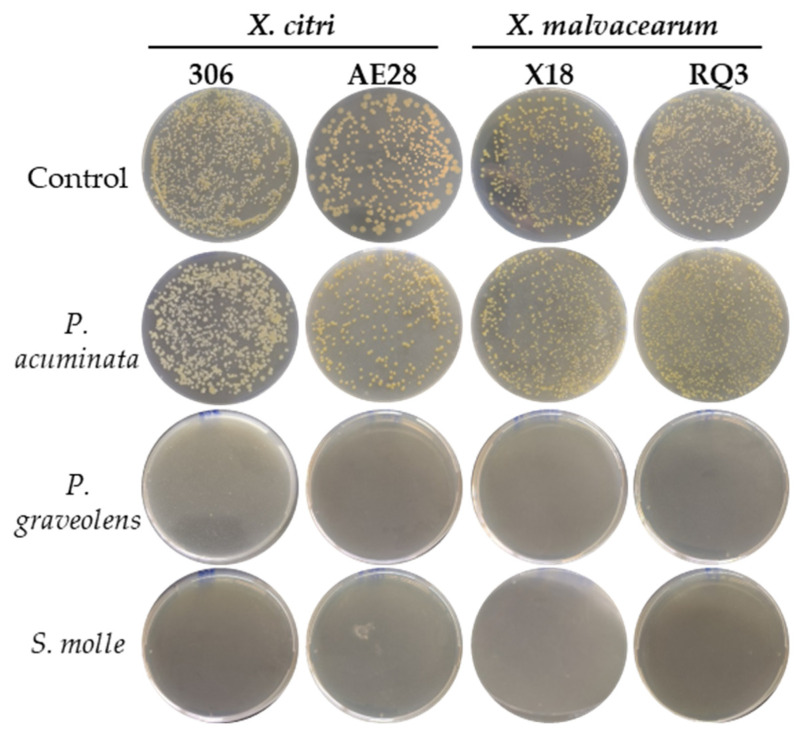
Effect of *P. acuminata* ethyl acetate extract, *P. graveolens*, and *S. molle* essential oils on in vitro growth of *X. citri* subsp. *citri* and *X. citri* subsp. *malvacearum* strains. Representative plates are shown for each treatment. The assay was conducted in triplicate.

The antibacterial activities of the plant-derived natural products were studied against *X. citri* and *X. malvacearum* strains (Table 2). The *P. acuminata* ethyl acetate extract did not significantly inhibit bacterial growth compared to the controls, whereas the essential oils obtained from *P. graveolens* and *S. molle* completely suppressed colony formation after 72 h of incubation (Figure 2; Supplementary Materials, Table S1). Notably, the response was consistent between regional and reference strains of *X. citri* and *X. malvacearum*. Consequently, local isolate and essential oils were selected for further in vivo assays on cotton bacterial blight disease.

### 2.4. Effects of S. molle and P. graveolens Essential Oils on Citrus Canker and Cotton Bacterial Blight Disease

To investigate whether essential oils derived from *P. graveolens* and *S. molle* could control *Xanthomonas* diseases, lemon leaves and cotton cotyledons were inoculated with regional AE28 and RQ3 strains, and two different in vivo assays were performed. The tissue was treated with essential oils 24 h before inoculation (preventive treatment) and 24 h after inoculation (curative treatment).

**Figure 3:**
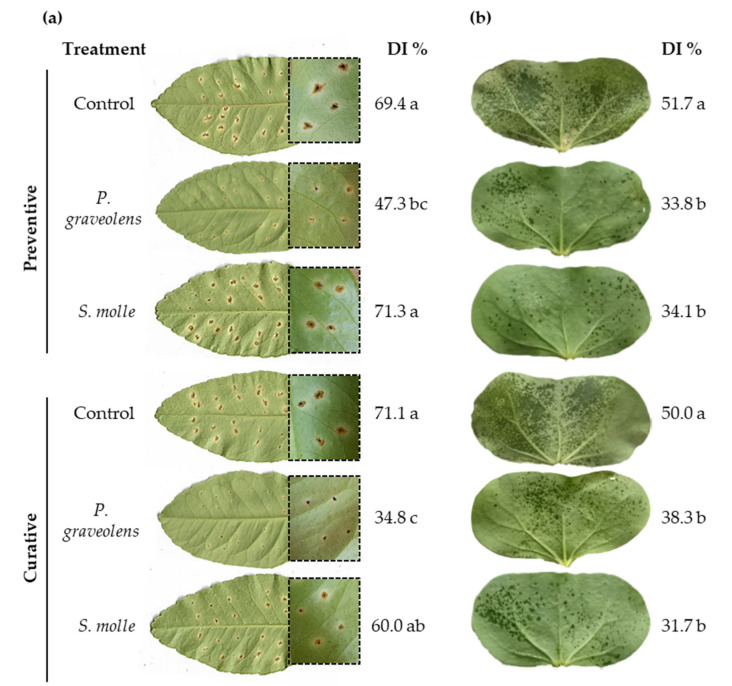
Antibacterial activity of essential oils on citrus canker (**a**) and cotton bacterial blight (**b**) disease severity at 20 days post-inoculation. Citrus leaves and cotton cotyledons were treated with *P. graveolens* and *S. molle* essential oils 24 h before (preventive) and after (curative) bacterial inoculation with *X. citri* subsp. *citri* AE28 and *X. citri* subsp. *malvacearum* RQ3. Disease index (DI %) values are expressed as means obtained from five lemon leaves and ten different cotton plants. Letters indicate significant differences at *p*-value < 0.05 (Tukey’s test). Distilled water treatment was used as a control.

Disease evaluation revealed distinct effects of essential oils on citrus canker and bacterial blight. Both preventive and curative treatments with *P. graveolens* significantly reduced citrus canker severity, while *S. molle* oil showed no significant reduction compared to the control (Figure 3a). In contrast, both essential oils led to a reduction in bacterial blight severity in cotyledons (Figure 3b). Notably, regardless of the timing of treatment, disease severity was reduced by approximately 1.5-fold compared to the control.

### 2.5. Phytochemical Profile of Active Natural Products

The essential oils were characterized based on the presence of compounds that represented more than 1% of their composition. Regarding the analysis of the chromatograms obtained by GC-MS, the following findings were determined.

The essential oil of *P. graveolens* was characterized by the presence of geraniol (24.89%), citronellol (19.5%), β-linalool (10.92%), γ-eudesmol (8.93%), citronellyl formate (6.30%), isomenthone (3.73%), geranyl tiglate (3.3%), D-germacrene (2.55%), and geranyl formate (2.14%), representing 82.26% of the total composition of the essential oil.

**Figure 4:**
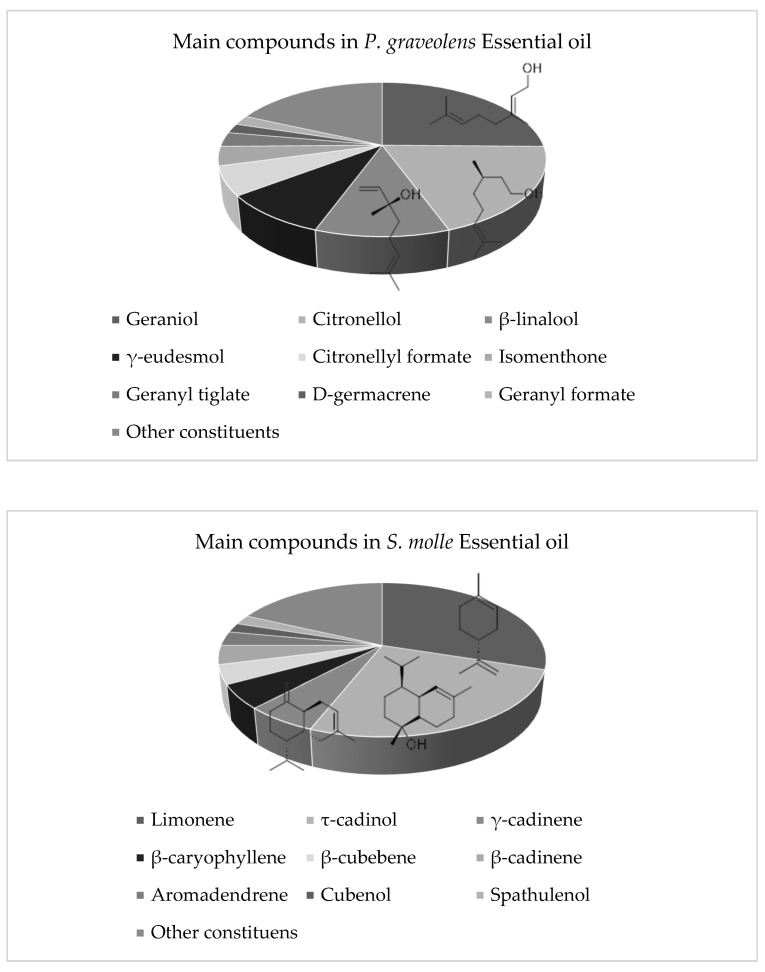
Percentages of the main volatile components and chemical structure of the three main components of *P. graveolens* and *S. molle* essential oils.

In the essential oil of *S. molle*, the presence of limonene (30.4%), τ-cadinol (25.9%), γ-cadinene (6.31%), β-caryophyllene (4.85%), β-cubebene (4.39%), β-cadinene (4.33%), aromadendrene (3.21%), cubenol (1.93%), spathulenol (1.93%), α-cadinol (1.76%), alloaromadendrene oxide-(2) (1.62%), and α-muurolene (1.09%) were detected, which together accounted for 89.16% of the total components. The chemical structures and the percentages of the main volatile components of the active essential oils are shown in Figure 4.

## 3. Discussion

Bacteria of the genus *Xanthomonas* are responsible for numerous plant diseases with significant economic and environmental impacts on agriculture and global trade. Accurate identification of *Xanthomonas* strains in a region is crucial for implementing effective control measures. In Argentina, the causal agent of cotton bacterial blight was previously identified based on morphological characteristics; however, molecular identification has not been conducted until now. In this study, the amplification of five housekeeping genes allowed the precise identification of *X. citri* subsp. *malvacearum* as the bacterial pathogen responsible for infecting cotton in northern Argentina.

Given the challenges of managing *Xanthomonas* infections, particularly in citrus and cotton, integrated management relies on a combination of cultural, chemical, and genetic strategies to reduce disease incidence and spread. However, in citrus, the prolonged use of copper-based treatments has raised significant ecological concerns, including soil accumulation, phytotoxicity to plant tissues, and the emergence of copper-resistant *Xanthomonas* populations [10]. In cotton, despite the availability of resistant genotypes, new *X. malvacearum* races have evolved and reemerged, posing ongoing challenges for disease control [15,16,17]. These limitations highlight the urgent need for more sustainable alternatives. In this context, plant-derived natural products represent a promising strategy for managing *Xanthomonas*, offering advantages such as environmental sustainability, diverse modes of action, and lower toxicity compared to conventional chemical pesticides. It has been demonstrated that hexane extracts of *Pterodon pubescens* seeds and *Psidium myrtoides* leaves and pericarp of unripe fruit exhibited promising activities against *X. citri* in vitro [30,31]. Additionally, aqueous extracts of *Mentha piperita*, *Syzygium cumini*, *Citrus limon*, *Moringa oleifera*, and *Syzygium aromaticum* showed promising results for *X. malvacearum* growth inhibition [20]. In citrus plants, foliar application of *Salvia rosmarinus* aqueous extracts significantly reduced the number of canker lesions when leaves were challenged with *X. citri* [32].

So far, there has been limited research on the natural products targeting these bacteria and their potential for application in crops. In the present study, we provide new insights into the in vitro and in vivo activity of three botanical-based products against *X. citri* and *X. malvacearum*, contributing to the development of more sustainable disease management strategies.

The native Argentine herb *P. acuminata* (syn. *Polygonum acuminatum*) has previously been reported for its antifungal properties against human pathogens, including *Candida albicans*, *C. tropicalis*, and *Cryptococcus neoformans*, and dermatophytes such as *Trichophyton mentagrophytes*, *T. rubrum*, and *Microsporum canis* [33]. It has also shown activity against phytopathogens like *Monilinia fructicola*, *Penicillium digitatum*, and *P. italicum* [23]. Three sesquiterpenes (polygodial, drimenol, and confertifolin) were isolated from the most active extract as the bioactive constituents. It was found that plants collected in March contained the highest concentration of these bioactive compounds [34]. In the present study, plants were collected in March 2024 from the same location as the previous study. The extract yield was similar to that of earlier work (1.8% now compared to 2.1% previously) [23,33,34]. However, the evaluation of *P. acuminata* AcOEt extract against different isolates of *X. citri* revealed no significant antibacterial activity. This suggests that, while the extracts and active compounds of *P. acuminata* may hold potential as fungicides [23,33,34], they are not effective as bactericides for controlling bacterial crop diseases. These findings are crucial for guiding future research and optimizing the use of *P. acuminata* in crop disease management.

On the other hand, the essential oil of *P. graveolens* was characterized by the presence of oxygenated monoterpenes such as geraniol, citronellol, β-linalool, and γ-eudesmol, among others. The yield and the main fraction of citronellol (35.2%) and geraniol (28.8%) in the oil obtained here coincides with findings by other authors [35,36]. Geraniol exhibited synergistic bactericidal activity when combined with chloramphenicol, norfloxacin, and tetracycline against *Klebsiella pneumoniae*, *Pseudomonas mirabilis*, *P. aeruginosa*, and *Staphylococcus aureus* [37]. Citronellol, which only differs from geraniol by having a double bond in its structure, also showed antifungal activity, inhibiting conidial germination and mycelium production of dermatophytes involving the inhibition of ergosterol biosynthesis [38].

In concordance with our study, Kačániová et al. examined the chemical profile using GC-MS, and 99.2% of the volatile compounds in *P. graveolens* essential oil were identified, with β-citronellol (29.7%) and geraniol (14.6%) being the most predominant; they showed strong antioxidant potential, particularly in neutralizing the ABTS radical cation. Antimicrobial tests revealed high effectiveness against biofilm-forming *Salmonella enterica* and *Priestia megaterium*. They also demonstrated significant antibiofilm activity, disrupting the biofilm of *S. enterica* on plastic and stainless steel [39]. *P. graveolens* essential oil in vitro fungicidal assay completely inhibited the growth of the phytopathogen *Botrytis cinerea* isolated from infected rose flowers; and the in vivo assay responded to the treatment by showing a significantly lower disease severity than those treated with commercial carbendazim [25], thus ensuring the effectiveness of this product for the treatment of *Botrytis*. Our results demonstrated that this essential oil also has the potential to inhibit *X. citri* bacterial growth in vitro and significantly reduce citrus canker and cotton bacterial blight in vivo. Moreover, effective control of the disease was independent of the timing of the natural product application (preventive and curative antibacterial effect).

For the essential oil of *S. molle*, the literature reports a high percentage of monoterpene hydrocarbons, among which are α- and β-pinene, myrcene, limonene, p-cymene, α- and β-phellandrene, and sabinene [40,41,42]. Although the major component of this oil obtained here is one of the expected for the species, the remaining major components involve oxygenated groups or sesquiterpene skeletons, which, although they appear in the cited literature, are found in minor compounds or traces. The two main compounds (limonene and τ-cadinol), comprising more than 50% of the mixture, have been reported to have strong antifungal activity [43,44,45]. In a recent study, da Silva et al. [46] demonstrated the in vitro antibacterial potential of *S. molle* essential oil and the main component, spathulenol, against *X. citri*; however, in vivo studies were not conducted to confirm the applicability for citrus biocontrol. Moreover, research on the antibacterial potential of the essential oils remains limited, particularly regarding their application in plant health. In our study, this essential oil demonstrated 100% inhibition of in vitro growth of two key *Xanthomonas* subspecies. Furthermore, applying it led to a 1.5-fold reduction in the severity of cotton bacterial blight, compared to the untreated controls; however, no significant reduction was observed for citrus canker. This differential behavior could be explained by differences in the *Xanthomonas* species involved, host plants, and the inoculation method.

Overall, our findings highlight the potential of plant-derived essential oils for managing *Xanthomonas* infections. Further research is needed to optimize their application and assess long-term effectiveness in field conditions, contributing to sustainable disease management.

## 4. Materials and Methods

### 4.1. Plant Material and Natural Products Obtaining

**Figure 5:**
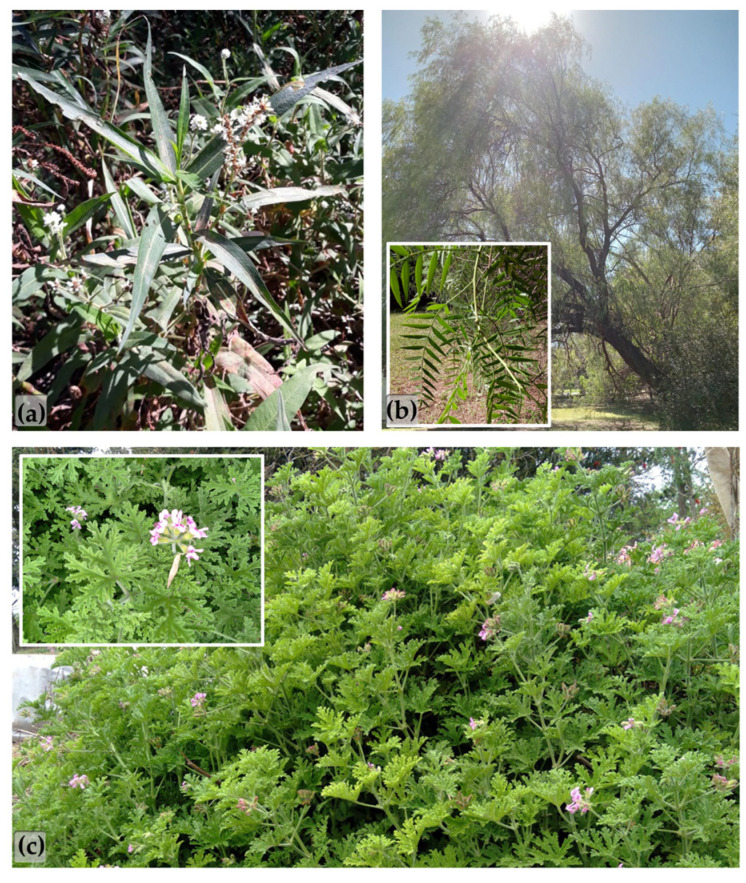
Pictures of the species in their natural environment and their parts used for the study: (**a**) *P. acuminata*, (**b**) *S. molle*, and (**c**) *P. graveolens*.

The plants were collected on the 19th of March 2024 between 10 and 12 am, during the end of the summer. A sample of each one was identified, and a *Voucher Specimen* was deposited in the FCA-UNL Herbarium “Arturo Ragonese” (Herbario SF), Kreder 2805-(3080HOF)-Esperanza, Argentina. Figure 5 shows pictures of the species in their natural environment and their parts used for the study. A dry extract was obtained from *P. acuminata* (Kunth) M. Gómez and two essential oils were obtained from *S. molle* L. and *P. graveolens* L’Hér. Healthy leaves of *P. acuminata* were dried, ground, and extracted (24 h × 3 cycles at room temperature with constant shaking) using ethyl acetate as solvent. The extracted solution was filtered to remove plant residues and concentrated under reduced pressure until there were no solvents left. In the case of essential-oil-producing plants (*S. molle* and *P. graveolens*), their fresh aerial parts underwent steam distillation. Yields were calculated based on 100 g of dry or fresh plant material, as appropriate.

### 4.2. Bacterial Strains, Culture Media, and Growth Conditions

*X. citri* and *X. malvacearum* regional isolates with confirmed pathogenicity as well as globally recognized reference strains were used in this work (Table 2). *X. citri* strain AE28 was isolated from an experimental field of the Universidad Nacional del Litoral (31°24′ S; 60°54′ W) [28]. *X. malvacearum* strain RQ3 was isolated from diseased cotton leaves collected from the experimental field of Instituto Nacional de Tecnología Agropecuaria (29°15′ S; 59°44′ W). All isolates were stored in 20% (*w*/*v*) glycerol at −80 °C until use, at which point they were cultured on Petri dishes with solid NYGB medium (bacteriological tryptone, 5 g/L; yeast extract, 3 g/L; glycerol, 20 g/L) at 28 °C. Bacteria were transferred to liquid NYGB medium at 28 °C with shaking at 200 rpm to prepare the suspensions. Overnight-saturated cultures were centrifuged at 7500 rpm for 10 min. The bacterial population was determined by measuring optical density (OD) at 600 nm in a spectrophotometer (721 model, BioTraza, Guangzhou, China) and adjusted at a concentration of 10^3^ or 10^7^ CFU/mL in 10 mM MgCl_2_ for in vitro and in vivo assays, respectively.

### 4.3. Molecular Identification of X. citri Regional Strains

The identity of *X. citri* and *X. malvacearum* regional strains used in this study were confirmed through molecular characterization. DNA bacteria extraction was performed following Chiesa et al. (2013) [13] and used as the template for PCR amplification. Molecular identification of *X. citri* AE28 was performed using *xpsD* primers, as previously described in Chiesa et al., 2013 [13]. *X. malvacearum* RQ3 identity was evaluated by using five housekeeping genes (*fusA*, *gapA*, *gltA*, *lacF*, *and lepA*) described by Almeida et al. (2010) [47]. PCR was performed on a T100 Thermal Cycler (Bio-Rad, Hercules, CA, USA) in 20 µL volume reaction containing 1× PCR buffer, 2.5 mM MgCl_2_, 1 µM each primer, 0.4 mM dNTPs, 1 U of Taq DNA polymerase (PB-L, Productos Bio-Lógicos (R), Buenos Aires, Argentina), and 50 ng of genomic DNA. Amplification was carried out with an initial denaturation step at 95 °C for 5 min, followed by 35 cycles each consisting of 95 °C for 1 min, 57 °C for 1 min, and 72 °C for 1 min, and a final extension of 72 °C for 10 min. PCR products were visualized on 1.5% (*w*/*v*) agarose gel and then sequenced with the same primers in Macrogen (Seoul, Republic of Korea). The obtained sequences were compared with those available in the GenBank Nucleotide Database (NCBI, https://www.ncbi.nlm.nih.gov/ accessed on 6 February 2025) using BLASTn (Nucleotide Basic Local Alignment Search Tool, version number 2.16.0, available since August 2021). All sequences generated in this study were deposited in GenBank.

### 4.4. In Vitro Susceptibility Test

For the bacterial inhibition assay with essential oils, 6 cm diameter glass Petri dishes were covered with 15 mL of previously melted NYGB medium. Once solidified, 50 µL of bacterial inoculum at a concentration of 10^3^ CFU/mL was placed at the center of the plate and spread in the medium with a Drigalsky spatula. After the inoculation solution evaporated, 15 μL of each essential oil or water (control experiment) was placed at the center of the Petri dish lid. This quantity of essential oil corresponds to a concentration of 1000 ppm, calculated based on the Petri dish dimensions. The prepared plates were incubated upside down so that the evaporating essential oil came into contact with the medium where the bacteria were growing. For fixed extracts of *P. acuminata*, the procedure was the same, but the extract solution was added to the culture medium before solidification to achieve the same final concentration as for the essential oils (1000 ppm).

The number of bacterial colonies grown in each plate was assessed on a colony counter (J-2, Hinotek) after 72 h of incubation at 28 °C, and compared with the control to estimate the percentage of inhibition. The assays were conducted in triplicate.

### 4.5. Preventive and Curative Disease Control with Essential Oils

Bioassays were conducted using cotton plants (BGSP166 genotype from INTA cotton germplasm bank) and one-year old lemon plants (Eureka genotype grafted onto *Poncirus trifoliata* rootstock). The hosts were treated with *S. molle* and *P. graveolens* essential oil at a final concentration of 1000 ppm, which had not caused phytotoxicity in a previous assay (see Supplementary Materials). Bacterial suspensions of *X. citri* AE28 and *X. malvacearum* RQ3 were inoculated using the pin-prick and spray methods on fully-expanded lemon leaves and cotton cotyledons, respectively.

For preventive treatment, lemon leaves and cotton cotyledons were sprayed with natural products or distilled water (as control) using a hand sprayer. Twenty-four hours later, the treated leaves were inoculated with *X. citri* and *X. malvacearum* bacterial suspensions. For curative treatment, the same procedure was used, except that the plants were initially inoculated with bacterial suspensions and 24 h later treated with natural products or distilled water. Plants were maintained for 20 days in a greenhouse, with a temperature range between 25 and 28 °C. Citrus canker severity was assessed by monitoring the progression of lesions from the pin-prick point (Supplementary Materials, Figure S2) and calculated using the following formula: Disease Index (DI %) = ∑ (scale grade × frequency) × 100/(total number of pin-prick lesions per leaf × maximum scale) [48]. Each treatment was applied to five lemon leaves, with 24 pin-prick lesions on each leaf. Bacterial blight severity was recorded using a modified scale previously prescribed by Sheo Raj (1998) [49] (Supplementary Materials, Figure S2) and calculated using the following formula: DI % = ∑ (scale grade x frequency) × 100/(total number of cotyledons × maximum scale) [48]. Each treatment was repeated on 10 cotton plants.

### 4.6. Chemical Profile of the Active Natural Products

The active essential oils were analyzed using an Agilent 7890B Gas Chromatograph coupled with an Agilent 5977 Mass Spectrometer (Agilent Technologies, Santa Clara, CA, USA), with a HP-5MS UI column (30 m × 0.25 mm with 0.25 µm film thickness). The running conditions were as follows: Injector at 250 °C; column temperature: 160 °C held for 3 min, then ramped at 5 °C/min to 30 °C; run time: 31 min. Mass spectrometer: full SCAN: 50-400, injection volume: 1 μL, split: 1:20. To identify the main components, the mass spectra of the most abundant peaks were compared with the database available on the equipment (NIST Mass Spectral Search Program for the NIST/EPA/NIH Mass Spectra Library version 2.0 build 19 November 2000).

### 4.7. Statistical Analysis

Data were analyzed using one-way ANOVA statistical analysis and Tukey’s test was used to determine the difference between treatments.

## 5. Conclusions

Regional isolates of *Xanthomonas* species AE28 and RQ3 were obtained from characteristic lesions on *C. limon* and *G. hirsutum*, respectively. RQ3 was completely characterized and firstly reported in this article. Essential oils extracted by steam distillation from the freshly aerial parts of *P. graveolens* and *S. molle* exhibited complete (100%) inhibition of bacterial growth in vitro at a concentration of 1000 ppm whereas the *P. acuminata* ethyl acetate extract resulted inactive, as determined by diffusion tests. Preventive and curative treatments with *P. graveolens* essential oil significantly reduced citrus canker severity, whereas *S. molle* essential oil did not show a significant reduction compared to the control. In contrast, regardless of the treatment’s timing, both essential oils effectively reduced bacterial blight severity in cotton cotyledons by approximately 1.5-fold. This study demonstrates that the essential oils of *P. graveolens* and *S. molle* have great potential as biological agents for managing bacterial diseases in key crops, promoting more sustainable agricultural strategies. In conclusion, *P. graveolens* essential oil showed the best result among the three natural products assayed in this work, reducing significantly the diseases caused by *Xanthomonas* in citrus and cotton tissues.

## Figures and Tables

**Table 1:** Plant material collection data and natural product yields.

Plant Scientific Name	*Voucher Specimen*	Collection Data	Part Used	Natural Product Type	Yield (%)
*P. acuminata*	MD53 UNL	19 March 2024, Salado River, Las Colonias, Santa Fe.	Dried leaves	EtOAc extract	1.80
*P. graveolens*	MD58 UNL	26 May 2024, CECIF, Las Colonias, Santa Fe.	Freshly aerial parts	Essential oil	0.46
*S. molle*	MD57 UNL	15 April 2024, FAVE, Las Colonias, Santa Fe.	Freshly aerial parts	Essential oil	0.72

**Table 2:** *Xanthomonas* strains used in this study.

Species	Strain	Origin	Reference
*X. citri* subsp. *citri*	306, reference	Brazil	da Silva et al., 2002 [27]
*X. citri* subsp. *citri*	AE28	Esperanza, Santa Fe, Argentina	Favaro et al., 2014 [28]
*X. citri* subsp. *malvacearum*	X18, reference	Burkina Faso	Cunnac et al., 2013 [29]
*X. citri* subsp. *malvacearum*	RQ3	Reconquista, Santa Fe, Argentina	This study

## Data Availability

Data are contained within the article.

## Supplementary Materials

The following supporting information can be downloaded at: https://www.mdpi.com/article/10.3390/plants14060957/s1, Figure S1. Analysis of *Xanthomonascitri* subsp. *citri* (*X. citri*) strain AE28 by specific molecular marker. PCR of *xpsD* marker and 2% (wt/vol) agarose gel electrophoresis containing ethidium bromide. M: molecular DNA Ladder (1-kb Plus DNA; Invitrogen, Carlsbad, CA, USA). Figure S2. Severity scales developed for citrus canker and cotton bacterial blight evaluation. 0: no macroscopic symptoms, 1: slight, 2: moderate, 3: severe, 4: highly severe infection. Table S1. Effect of *Persicaria acuminata* extract and *Pelargonium graveolens* and *Schinus molle* essential oils on the in vitro growth of *Xanthomonas citri* subsp. *citri* and *Xanthomonas citri* subsp. *malvacearum*.

## References

[1] Citrus: World Markets and Trade. https://apps.fas.usda.gov/psdonline/circulars/citrus.pdf?utm_source.

[2] Khan M.A., Wahid A., Ahmad M., Tahir M.T., Ahmed M., Ahmad S., Hasanuzzaman M., Ahmad S., Hasanuzzaman M. (2020). World cotton production and consumption: An overview. Cotton Production and Uses: Agronomy, Crop Protection, and Postharvest Technologies.

[3] The Argentine Citrus Industry. https://www.federcitrus.org/wp-content/uploads/2024/11/Federcitrus-Actividad-Citricola-2024.pdf.

[4] Scarpin G.J., Dileo P.N., Winkler H.M., Cereijo A.E., Lorenzini F.G., Muchut R.J., Roeschlin R.A., Acuña C., Paytas M. (2025). Genetic progress in cotton dry matter partitioning in Argentina. Ind. Crop. Prod..

[5] An S.Q., Potnis N., Dow M., Vorhölter F.J., He Y.Q., Becker A., Teper D., Li Y., Wang N., Bleris L. (2020). Mechanistic insights into host adaptation, virulence and epidemiology of the phytopathogen *Xanthomonas*. FEMS Microbiol. Rev..

[6] Gabriel D., Gottwald T., Lopes S.A., Wulff N.A., Talon M., Caruso M., Fred G., Gmitter F.G. (2020). Bacterial pathogens of citrus: Citrus canker, citrus variegated chlorosis, and Huanglongbing. The Genus Citrus.

[7] Delannoy E., Lyon B., Marmey P., Jalloul A., Montillet J., Daniel J., Essenberg M., Nicole M. (2005). Resistance of cotton to *Xanthomonas campestris* pv. *malvacearum*. Annu. Rev. Phytopathol..

[8] Favaro M.A., Roeschlin R.A., Ribero G.G., Maumary R.L., Fernandez L.N., Lutz A., Sillon M., Rista L.M., Marano M.R., Gariglio N.F. (2017). Relationships between copper content in orange leaves, bacterial biofilm formation, and citrus canker disease control after different copper treatments. Crop Prot..

[9] Favaro M.A., Molina M.C., Roeschlin R.A., Gadea J., Gariglio N.F., Marano M.R. (2020). Different responses in mandarin cultivars uncover a role of cuticular waxes in the resistance to citrus canker. Phytopathology.

[10] Behlau F., Gochez A.M., Jones J.B. (2020). Diversity and copper resistance of *Xanthomonas* affecting citrus. Trop. Plant Pathol..

[11] Mačionienė I., Čepukoit D., Šalomskienė J., Černauskas D., Burokienė D., Šalaševičienė A. (2021). Effects of natural antimicrobials on *Xanthomonas* strains growth. Horticulturae.

[12] Zhang J., Elassbli H., Zhu Y., Wheeler T., Bourland F. (2024). Evaluation methods, resistant germplasm, and breeding for resistance to bacterial blight in cotton: A review. J. Cotton Sci..

[13] Chiesa M.A., Siciliano M.F., Ornella L., Roeschlin R.A., Favaro M.A., Delgado N.P., Sendín L.N., Orce I.G., Ploper L.D., Vojnov A.A. (2013). Characterization of a variant of *Xanthomonas citri* subsp. *citri* that triggers a host-specific defense response. Phytopathology.

[14] Roeschlin R.A., Favaro M.A., Chiesa M.A., Alemano S., Vojnov A.A., Castagnaro A.P., Filippone M.P., Gmitter F.G., Gadea J., Marano M.R. (2017). Resistance to citrus canker induced by a variant of *Xanthomonas citri* ssp. *citri* is associated with a hypersensitive cell death response involving autophagy-associated vacuolar processes. Mol. Plant Pathol..

[15] Huang X., Zhai J., Luo Y., Rudolph K. (2008). Identification of a highly virulent strain of *Xanthomonas axonopodis* pv. *malvacearum*. Eur. J. Plant Pathol..

[16] Phillips A.Z., Berry J.C., Wilson M.C., Vijayaraghavan A., Burke J., Bunn J.I., Bart R.S. (2017). Genomics-enabled analysis of the emergent disease cotton bacterial blight. PLoS Genet..

[17] Chavhan R.L., Mondal K.K., Karuppayil S.M., Chakrabarty P.K. (2021). Evolution of biotypes within race 18 population of *Xanthomonas citri* subsp. *malvacearum* and their predominance in Indian cotton belts. Physiol. Mol. Plant Pathol..

[18] Di Liberto M., Stegmayer M.I., Svetaz L., Derita M. (2019). Evaluation of Argentinean medicinal plants and isolation of their bioactive compounds as an alternative for the control of postharvest fruits phytopathogenic fungi. Br. J. Pharmacog..

[19] Basaid K., Chebli B., Mayad E.H., Furze J.N., Bouharroud R., Krier F., Paulitz T. (2021). Biological activities of essential oils and lipopeptides applied to control plant pests and diseases: A review. Int. J. Pest Manag..

[20] Naqvi S.A.H., Iqbal S., Farooq U., Hassan M.Z., Shahid M.N., Noor Shah A., Abbas A., Mubeen I., Farooq A., Ghareeb R.Y. (2022). Evaluation of bacterial perpetuation assays and plant biomolecules antimicrobial activity against cotton blight bacterium *Xanthomonas citri* subsp. *malvacearum*; an alternative source for food production and protection. Plants.

[21] Košćak L., Lamovšek J., Đermić E., Prgomet I., Godena S. (2023). Microbial and plant-based compounds as alternatives for the control of phytopathogenic bacteria. Horticulturae.

[22] Aslam M.N., Khaliq H., Zhao H., Moosa A., Maqsood A., Farooqi M.A., Bilal M.S., Mahmood T., Mukhtar T. (2025). Thymol as a Novel Plant-Derived Antibacterial Agent for Suppressing *Xanthomonas citri* pv. *malvacearum* in Cotton. Curr. Microbiol..

[23] Di Liberto M., Seimandi G., Fernández L., Ruiz V., Svetaz L., Derita M. (2021). Botanical control of citrus green mold and peach brown rot on fruits assays using a *Persicaria acuminata* phytochemically characterized extract. Plants.

[24] Stegmayer M.I., Fernández L., Alvarez N., Olivella L., Gutiérrez H., Favaro M.A., Derita M. (2021). Aceites esenciales provenientes de plantas nativas para el control de hongos fitopatógenos que afectan a frutales. Rev. FAVE-Cienc. Agrar..

[25] Stegmayer M.I., Alvarez N.H., Sager N., Buyatti M., Derita M.G. (2022). Evaluation of *Pelargonium graveolens* essential oil to prevent gray mold in rose flowers. J. Plant Prot. Res..

[26] Alvarez N., Stegmayer M.I., Seimandi G., Pensiero J.F., Zabala J.M., Favaro M.A., Derita M.G. (2023). Natural Products Obtained from Argentinean Native Plants Are Fungicidal against Citrus Postharvest Diseases. Horticulturae.

[27] da Silva A.R., Ferro J.A., Reinach F.D.C., Farah C.S., Furlan L.R., Quaggio R.B., Kitajima J.P. (2002). Comparison of the genomes of two *Xanthomonas* pathogens with differing host specificities. Nature.

[28] Favaro M.A., Micheloud N.G., Roeschlin R.A., Chiesa M.A., Castagnaro A.P., Vojnov A.A., Gmitter F.G., Gadea J., Rista L.M., Gariglio N.F. (2014). Surface barriers of mandarin ‘Okitsu’ leaves make a major contribution to canker disease resistance. Phytopathology.

[29] Cunnac S., Bolot S., Forero Serna N., Ortiz E., Szurek B., Noël L.D., Arlat M., Jacques M.A., Gagnevin L., Carrere S. (2013). High-quality draft genome sequences of two *Xanthomonas citri* pv. *malvacearum* strains. Genome Announc..

[30] dos Santos J., Fernandes C., Silva N., Calefi G., Martins C., Volpini G., Crotti A., Ribeiro A., Esperandim T., Tavares D. (2025). Volatile compounds of hexane extract from *Pterodon pubescens* Benth seeds and its significant in vitro potential against different bacterial strains. Nat. Prod. Res..

[31] Ribeiro A.M.R., Fernandes C.C., Menezes R.d.P., Oliveira A.M., Gonçalves D.S., Martins C.H.G., Miranda M.L.D. (2024). Antibacterial screening of hexane extracts from *Psidium myrtoides*, a Brazilian native plant. Ciência E Nat..

[32] Martin A.P., Martínez M.F., Chiesa M.A., Garcia L., Gerhardt N., Uviedo F., Torres P.S., Marano M.R. (2023). Priming crop plants with rosemary (*Salvia rosmarinus* Spenn, syn *Rosmarinus officinalis* L.) extract triggers protective defense response against pathogens. Plant Physiol. Biochem..

[33] Derita M., Montenegro I., Garibotto F., Enriz R., Cuellar Fritis M., Zacchino S. (2013). Structural Requirements for the Antifungal Activities of Natural Drimane Sesquiterpenes and Analogues, Supported by Conformational and Electronic Studies. Molecules.

[34] Derita M., Leiva M., Zacchino S. (2009). Influence of plant part, season of collection and content of the main active constituent, on the antifungal properties of *Polygonum acuminatum* Kunth. J. Ethnopharmacol..

[35] Babu K.G., Kaul V.K. (2013). Variation in essential oil composition of rose-scented geranium (*Pelargonium* sp.) distilled by different distillation techniques. Flav. Fragr. J..

[36] Bouzenna H., Krichen L. (2013). *Pelargonium graveolens* L’Her. and *Artemisia arborescens* L. essential oils: Chemical composition, antifungal activity against *Rhizoctonia solani* and insecticidal activity against *Rhysopertha dominica*. Nat. Prod. Res..

[37] Lira M.H.P.D., Andrade Júnior F.P.D., Moraes G.F.Q., Macena G.D.S., Pereira F.D.O., Lima I.O. (2020). Antimicrobial activity of geraniol: An integrative review. J. Essent. Oil Res..

[38] Pereira F.D.O., Mendes J.M., Lima I.O., Mota K.S.D.L., Oliveira W.A.D., Lima E.D.O. (2015). Antifungal activity of geraniol and citronellol, two monoterpenes’ alcohols, against *Trichophyton rubrum* involves inhibition of ergosterol biosynthesis. Pharm. Biol..

[39] Kačániová M., Vukic M., Vukovic N.L., Čmiková N., Verešová A., Schwarzová M., Garzoli S. (2023). An in-depth study on the chemical composition and biological effects of *Pelargonium graveolens* essential oil. Foods.

[40] Gomes V., Agostini G., Agostini F., Atti dos Santos A.C., Rossato M. (2013). Variation in the essential oils composition in Brazilian populations of *Schinus molle* L. (Anacardiaceae). Biochem. Syst. Ecol..

[41] Do Rosário Martins M., Arantes S., Candeias F., Tinoco M.T., Cruz-Morais J. (2014). Antioxidant, antimicrobial and toxicological properties of *Schinus molle* L. essential oils. J. Ethnopharmacol..

[42] Do Prado A.C., Garces H.G., Bagagli E., Rall V.L.M., Furlanetto A., Fernandes Junior A., Furtado F.B. (2019). *Schinus molle* essential oil as a potential source of bioactive compounds: Antifungal and antibacterial properties. J. Appl. Microbiol..

[43] Ho C.L., Liao P.C., Wang E.I.C., Su Y.C. (2011). Composition and antifungal activities of the leaf essential oil of *Neolitsea parvigemma* from Taiwan. Nat. Prod. Commun..

[44] Mahdavi Omran S., Moodi M.A., Norozian Amiri S.M.B., Mosavi S.J., Ghazi Mir Saeed S.A.M. (2011). The effects of limonene and orange peel extracts on some spoilage fungi. Int. J. Mol. Clin. Microbiol..

[45] Marei G.I.K., Rasoul M.A.A., Abdelgaleil S.A. (2012). Comparative antifungal activities and biochemical effects of monoterpenes on plant pathogenic fungi. Pestic. Biochem. Physiol..

[46] da Silva I.R.R., Fernandes C.C., Gonçalves D.S., Martins C.H.G., Miranda M.L.D. (2023). Chemical composition and anti-*Xanthomonas citri* activities of essential oils from *Schinus molle* L. fresh and dry leaves and of its major constituent spathulenol. Nat. Prod. Res..

[47] Almeida N.F., Yan S., Cai R., Clarke C.R., Morris C.E., Schaad N.W., Vinatzer B.A. (2010). PAMDB, a multilocus sequence typing and analysis database and website for plant-associated microbes. Phytopathology.

[48] McKinney H.H. (1923). Influence of soil temperature and moisture on infection of wheat seedlings by *Helminthosporium sativum*. J. Agric. Res..

[49] Buttar D., Pawar T., Grewal I. (2022). Impact of Priaxor (fluxapyroxad 167 g/L + pyraclostrobin 333 g/L SC) on fungal foliar leaf spots in upland cotton. Pl. Dis. Res..

