# Supplementary material for: Botanical-Based Strategies for Controlling Xanthomonas spp. in Cotton and Citrus: In Vitro and In Vivo Evaluation
Source: Plants (Basel). 2025 Mar 19;14(6):957. doi: 10.3390/plants14060957 (PMC11945062; doi:10.3390/plants14060957)
Supplement: Supplementary file 1 [file plants-14-00957-s001.zip › Supplementary Figure S1.pdf]

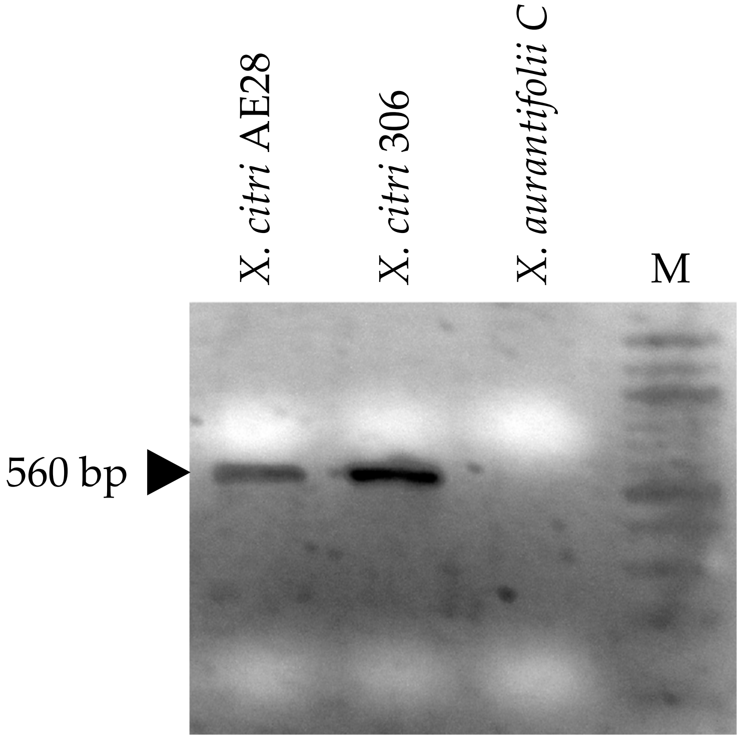

Supplemental Figure S1. Analysis of *Xanthomonas citri* subsp. *citri* (*X. citri*) strain AE28 by specific molecular marker. PCR of *xpsD* marker and 2% (wt/vol) agarose gel electrophoresis containing ethidium bromide. M: molecular DNA Ladder (1-kb Plus DNA; Invitrogen, Carlsbad, CA).
