# Supplementary material for: Botanical-Based Strategies for Controlling Xanthomonas spp. in Cotton and Citrus: In Vitro and In Vivo Evaluation
Source: Plants (Basel). 2025 Mar 19;14(6):957. doi: 10.3390/plants14060957 (PMC11945062; doi:10.3390/plants14060957)
Supplement: Supplementary file 1 [file plants-14-00957-s001.zip › Supplementary Figure S2.pdf]

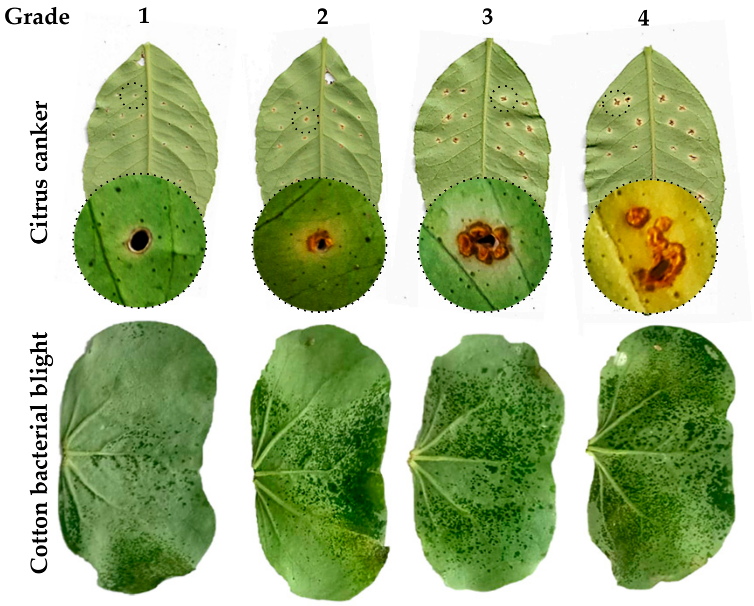

Supplementary Figure S2. Severity scales developed for citrus canker and cotton bacterial blight evaluation. 0: no macroscopic symptoms, 1: slight, 2: moderate, 3: severe, 4: highly severe infection.
