# Supplementary material for: Botanical-Based Strategies for Controlling Xanthomonas spp. in Cotton and Citrus: In Vitro and In Vivo Evaluation
Source: Plants (Basel). 2025 Mar 19;14(6):957. doi: 10.3390/plants14060957 (PMC11945062; doi:10.3390/plants14060957)
Supplement: Supplementary file 1 [file plants-14-00957-s001.zip › Supplementary Table S1.pdf]

Supplementary Table S1. Effect of *Persicaria acuminata* extract and *Pelargonium graveolens* and *Schinus molle* essential oils on the *in vitro* growth of *Xanthomonas citri* subsp. *citri* and *Xanthomonas citri* subsp. *malvacearum*.

**% Growth inhibition**	***X. citri* 306**	***X. citri* A28**	***X. malvacearum* X18**	***X. malvacearum* RQ3**
P. acuminata	**0 a**	**24 a**	**0 a**	**0 a**
P. graveolens	**100 b**	**100 b**	**100 b**	**100 b**
S. molle	**100 b**	**100 b**	**100 b**	**100 b**

Different letters within each column indicate significant differences ($P < 0.05$, Tukey's test).
